# Fault Diagnosis Method for Shearer Arm Gear Based on Improved S-Transform and Depthwise Separable Convolution

**DOI:** 10.3390/s25134067

**Published:** 2025-06-30

**Authors:** Haiyang Wu, Hui Zhou, Chang Liu, Gang Cheng, Yusong Pang

**Affiliations:** 1 School of Mechanical and Electrical Engineering, China University of Mining and Technology, Xuzhou 221116, China; ts22050183p31@cumt.edu.cn (H.W.);; 2 School of Chemical Engineering and Technology, China University of Mining and Technology, Xuzhou 221116, China; 3 School of Mechanical and Electrical Engineering, Xuzhou University of Technology, Xuzhou 221116, China; 4 Faculty of Mechanical Engineering, Delft University of Technology, 2628 CD Delft, The Netherlands

**Keywords:** shearer rocker arm, S-transform, depth separable convolution

## Abstract

To address the limitations in time–frequency feature representation of shearer arm gear faults and the issues of parameter redundancy and low training efficiency in standard convolutional neural networks (CNNs), this study proposes a diagnostic method based on an improved S-transform and a Depthwise Separable Convolutional Neural Network (DSCNN). First, the improved S-transform is employed to perform time–frequency analysis on the vibration signals, converting the original one-dimensional signals into two-dimensional time–frequency images to fully preserve the fault characteristics of the gear. Then, a neural network model combining standard convolution and depthwise separable convolution is constructed for fault identification. The experimental dataset includes five gear conditions: tooth deficiency, tooth breakage, tooth wear, tooth crack, and normal. The performance of various frequency-domain and time-frequency methods—Wavelet Transform, Fourier Transform, S-transform, and Gramian Angular Field (GAF)—is compared using the same network model. Furthermore, Grad-CAM is applied to visualize the responses of key convolutional layers, highlighting the regions of interest related to gear fault features. Finally, four typical CNN architectures are analyzed and compared: Deep Convolutional Neural Network (DCNN), InceptionV3, Residual Network (ResNet), and Pyramid Convolutional Neural Network (PCNN). Experimental results demonstrate that frequency–domain representations consistently outperform raw time-domain signals in fault diagnosis tasks. Grad-CAM effectively verifies the model’s accurate focus on critical fault features. Moreover, the proposed method achieves high classification accuracy while reducing both training time and the number of model parameters.

## 1. Introduction

As a core component of the transmission system, the shearer arm gear’s health directly affects the operational stability of the equipment. However, under high load, severe impacts, and complex working conditions, the gear is prone to faults such as wear, pitting, and fractures, potentially causing equipment shutdowns or even severe safety accidents [1]. Therefore, accurately extracting gear fault features in complex environments and improving diagnostic accuracy remain critical challenges in the field of intelligent fault diagnosis.

In recent years, various gear fault diagnosis methods based on time–frequency analysis and deep learning have been proposed. Time–frequency analysis techniques effectively extract features from non-stationary signals, including Short-Time Fourier Transform (STFT) [2,3,4], Wavelet Transform (WT) [5,6], Empirical Mode Decomposition (EMD) [7,8], and Variational Mode Decomposition (VMD) [9,10]. However, STFT is limited by a fixed window size, making it difficult to balance time–frequency resolution. The choice of mother wavelet in WT is subjective, while both EMD and VMD are susceptible to mode mixing.

To address the aforementioned issues, an increasing number of studies have introduced deep learning into fault diagnosis tasks [11]. For example, Huang et al. combined convolutional neural networks (CNNs) with Wavelet Transform (WT) for feature extraction and classification [12]. However, CNNs typically demand high computational resources, limiting their suitability for edge device deployment. Yin et al. constructed a long short-term memory (LSTM) network to capture temporal dependencies in time-series data, which improved recognition of long-duration signals [13]. Nevertheless, the training process of LSTM is prone to gradient vanishing, leading to a performance drop of over 12% in complex datasets. To reduce dependence on labeled data, Chen et al. proposed an unsupervised fault diagnosis approach based on autoencoders (AE) [14]. However, its performance fluctuates significantly (up to ± 15%) under small sample sizes or strong noise interference. Qiu et al. integrated variational mode decomposition (VMD) with ResNet for complex operating conditions [15], but the model’s structural complexity led to high computational demands [16,17].

To enable efficient fault detection under resource-constrained scenarios, Li et al. proposed a rotating machinery fault diagnosis method based on MobileNet, which reduced the number of parameters by approximately 75% and achieved millisecond-level inference latency [18]. Ren et al. developed a lightweight diagnostic framework by incorporating multi-stage pruning and knowledge distillation, significantly reducing the model size while maintaining accuracy comparable to ResNet [19]. However, most of these methods primarily focus on static fault signals and perform well under low-noise conditions, whereas their effectiveness under noisy environments remains limited.

Against this backdrop, enhancing time–frequency resolution and noise robustness during feature extraction has become a key challenge to be addressed. Chen et al. employed the short-time Fourier Transform (STFT) to generate time–frequency representations and integrated it with a CNN for classification, achieving end-to-end automatic feature extraction [20]. However, due to the use of a fixed window function in STFT, the diagnostic accuracy decreased by approximately 6% to 9% when dealing with complex signals. To mitigate this issue, Li et al. proposed optimizing the Gaussian window structure in S-transform to improve the time–frequency localization of transient features [21]. Nevertheless, the window parameters still rely on manual configuration, limiting adaptability. Kazemi et al. introduced a noise suppression strategy prior to transformation to enhance the saliency of principal components in the time–frequency representation and reduce noise interference [22]. Although this method improved feature stability, it heavily depended on preprocessing algorithms and exhibited limited robustness under strong impact conditions.

To address the limitations of the aforementioned studies, this paper proposes a fault diagnosis method that integrates an adaptively modulated Gaussian-windowed S-transform with a depthwise separable convolutional neural network (DSCNN). First, the method employs a local energy-based adaptive mechanism to dynamically adjust the window parameters of the S-transform, effectively overcoming the resolution limitations when processing non-stationary signals and enabling the adaptive extraction of key features. Then, the standard convolution in CNN is replaced by a depthwise separable convolution, which decomposes the operation into depthwise and pointwise convolutions for spatial feature extraction and channel-wise fusion, respectively. This replacement significantly reduces computational cost and model complexity, thereby improving diagnostic efficiency. The main contributions of this study are summarized as follows:

(1) A diagnostic framework combining adaptive window S-transform and depthwise separable convolutional networks is proposed. Compared with existing methods using fixed-window S-transform or complex convolutional architectures, the proposed approach can dynamically adapt to energy variations in different fault signals while effectively reducing model complexity.
(2) A window-width adjustment mechanism based on the energy distribution of the signal is designed, enabling more accurate time–frequency decomposition of transient fault signals and enhancing diagnostic robustness.
(3) A lightweight diagnostic network architecture is developed, which, compared to deep models such as ResNet, achieves a reduction of approximately 66% in parameter count and nearly 12% in inference latency per prediction, while maintaining comparable diagnostic accuracy—demonstrating its potential for deployment in edge computing scenarios.

## 2. Method for Fault Diagnosis

### 2.1. Theory of the Improved S-Transform

The S-transform integrates the advantages of short-time Fourier Transform and Wavelet Transform, offering flexible time–frequency resolution [23,24,25]. However, under noisy conditions, the standard S-transform adopts a fixed window width, which may lead to time–frequency smearing. To enhance its noise robustness and time–frequency concentration, this study introduces an adaptive Gaussian window mechanism based on the local energy of the signal, aiming to improve the stability of feature extraction. The standard definition of the S-transform is as follows:

(1) $$S(t,f)=\int_{-\infty }^{\infty }x(\tau )\cdot \exp\left(-\frac{{\left(t-\tau \right)}^{2}}{2\sigma^{2}}\right)\cdot e^{-j2\pi f\tau }d\tau$$

In this expression, x(τ) denotes the input signal, t and f represent the time and frequency variables, respectively, and σ is the standard deviation of the Gaussian window.

To achieve adaptive window-width adjustment, the window width σ is redefined as a time-dependent function σ(t), which is expressed as

(2) $$\sigma (t)=\frac{k}{\sqrt{E(t)}}$$

In this expression, k is a scaling coefficient, and E(t) represents the local energy of the signal around time t. It is calculated as follows:

(3) $$E(t)=\int_{t-\Delta }^{t+\Delta }|x(\tau ){|}^{2}d\tau$$

In this expression, Δ denotes half the width of the window used for local energy calculation.

The improved S-transform (IST) is defined as

(4) $$S_{\mathrm{adaptive}}(t,f)=\int_{-\infty }^{\infty }x(\tau )\cdot \exp\left(-\frac{{\left(t-\tau \right)}^{2}}{2\sigma^{2}(t)}\right)\cdot e^{-j2\pi f\tau }d\tau$$

In this expression, the adaptive window width σ(t) is dynamically adjusted, according to the local energy variation.

### 2.2. Depthwise Separable Convolution

Depthwise separable convolution is a convolutional operation designed to reduce computational complexity while enhancing feature extraction capability [26,27,28]. It decomposes the standard convolution into two steps: depthwise convolution and pointwise convolution, significantly reducing the amount of computation while preserving the model’s representational power.

The computational complexity of a standard CNN convolution is given by

(5) $$O(WHC_{\mathrm{in}}C_{\mathrm{out}}KK)$$

While the computational complexity of DSCNN is given by

(6) $$O(WHC_{\mathrm{in}}KK)+O(WHC_{\mathrm{in}}C_{\mathrm{out}})$$

In this expression, W and H represent the width and height of the feature map, $C_{\mathrm{in}}$ and $C_{\mathrm{out}}$ denote the numbers of input and output channels, respectively, and K is the size of the convolution kernel.

### 2.3. Construction of the Diagnostic Model

**Figure 1:**
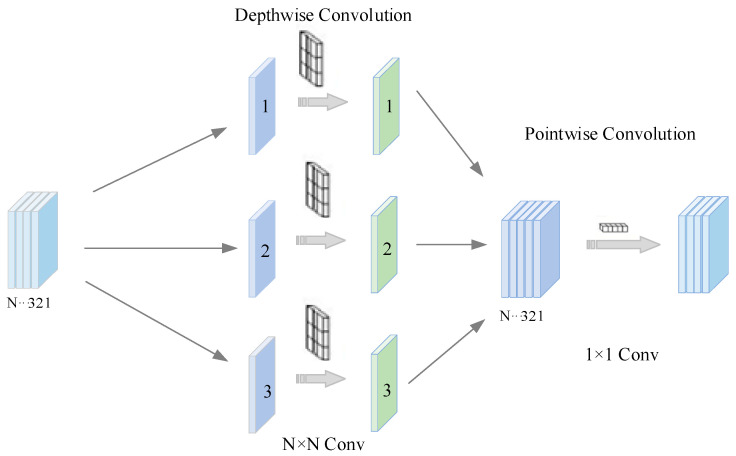
Depthwise separable convolution structure.

A DSCNN architecture was constructed to achieve efficient and accurate gear fault classification. The model takes 64 × 64 × 3 two-dimensional time–frequency images as input and enhances computational efficiency and classification performance through layer-by-layer feature extraction and a depthwise separable convolution strategy. Initially, the input data passes through a convolutional layer to extract low-level features. This layer employs batch normalization to accelerate convergence and reduce internal covariate shift, while the ReLU activation function is used to enhance nonlinear representation capability. Next, max pooling is applied for dimensionality reduction, which decreases computational load while preserving critical information. In the deeper layers of the network, the model gradually increases the number of channels and performs multi-level feature extraction through Conv2, Conv3, Conv4, and Conv5 layers. Each layer is equipped with batch normalization and ReLU activation to enhance stability and representation capability. Notably, Conv3 and Conv5 adopt pointwise convolution to efficiently integrate channel information while maintaining spatial structure. The depthwise separable convolution mechanism is illustrated in Figure 1. After feature extraction, the model performs fault classification via fully connected layers, followed by a Softmax layer for output normalization. Finally, the classification layer computes the loss to enable end-to-end training. Compared with standard convolutional neural networks, this model not only ensures high classification accuracy, but also significantly reduces computational complexity through depthwise separable convolutions, thereby improving both training and inference speed.

### 2.4. Fault Diagnosis Process

**Figure 2:**
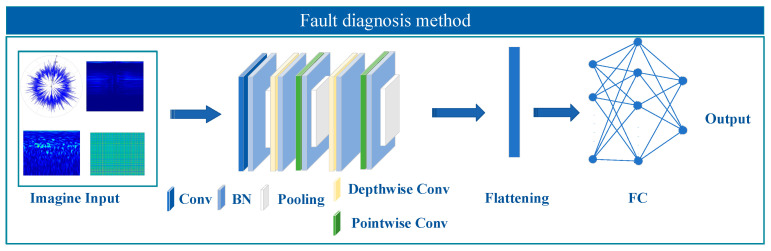
Fault diagnosis process of the shearer arm gearbox.

This paper proposes a fault diagnosis method for gear vibration signals under various fault types, based on an IST and a DSCNN. The approach first preprocesses the vibration signals and simulates complex operating conditions by adding noise at different signal-to-noise ratios to enhance model robustness. Then, the improved S-transform is applied for time–frequency analysis, with an optimized adaptive window function that adjusts time and frequency resolution to enhance critical time–frequency information and improve feature separability. On this basis, a DSCNN is constructed for feature extraction and classification, fully exploiting the local correlations of time–frequency features to improve the model’s ability to recognize different fault patterns. Experimental results demonstrate that the proposed method maintains stable diagnostic accuracy under various noise levels and outperforms standard methods in terms of computational efficiency and feature extraction capability. Figure 2 illustrates the fault diagnosis process for the shearer arm gear.

## 3. Experimental Data Acquisition

**Figure 3:**
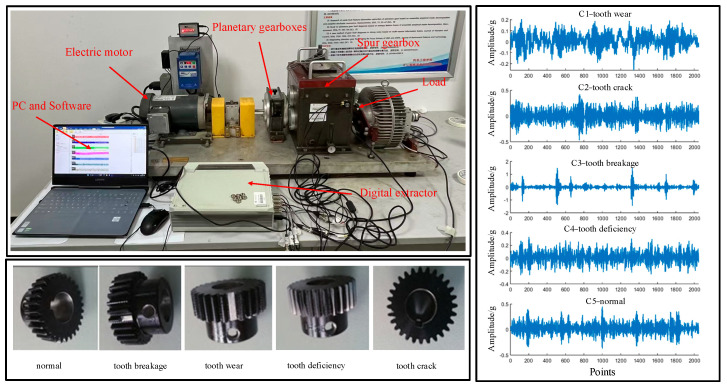
Power transmission fault diagnosis and signal acquisition platform.

To validate the proposed method, this study uses gear vibration data collected from the Drivetrain Diagnosis Simulation (DDS) testbed at China University of Mining and Technology. As shown in Figure 3, the test rig is composed of a two-stage planetary gearbox, a two-stage parallel-axis gearbox, a programmable magnetic powder brake (load range: 1.5–32 ft·lb), a 3-horsepower AC motor (variable speed up to 5000 rpm), a torque sensor (range: 20 N·m, integrated with a 360-pulse encoder), and various vibration sensors mounted on bearing housings and gearboxes. The system allows the introduction of both single and coupled faults in the gear and bearing components, and supports the application of torsional and radial loads to simulate real operational stresses.

In this study, the experimental configuration involved setting the motor speed to 1800 rpm (equivalent to 30 revolutions per second, r/s), applying a magnetic brake torque of 20 N·m at the output shaft, and using a sampling frequency of 12,800 Hz with a total sampling duration of 20 s per trial. The sampling frequency of 12,800 Hz was selected to ensure accurate capture of high-frequency components related to gear fault characteristics, avoiding aliasing and preserving diagnostic information.

The DDS rig enables the simulation of various fault types, including the following: (i) normal, (ii) tooth breakage (complete loss of one tooth), (iii) tooth crack (initiation of a fatigue crack at the gear root), (iv) tooth wear (uniform surface material loss), and (v) tooth deficiency (intentional removal of teeth to simulate backlash or undercut).

To enhance the stationarity and analyzability of the vibration signals, a sliding smoothing technique was applied for signal pre-processing. Each signal segment contains 2048 data points, with a sliding window size of 1024. A total of 200 signal samples were acquired for each fault type, resulting in a dataset of 1000 labeled gear fault samples.

## 4. Experimental Analysis

### 4.1. Preprocessing Analysis

**Figure 4:**
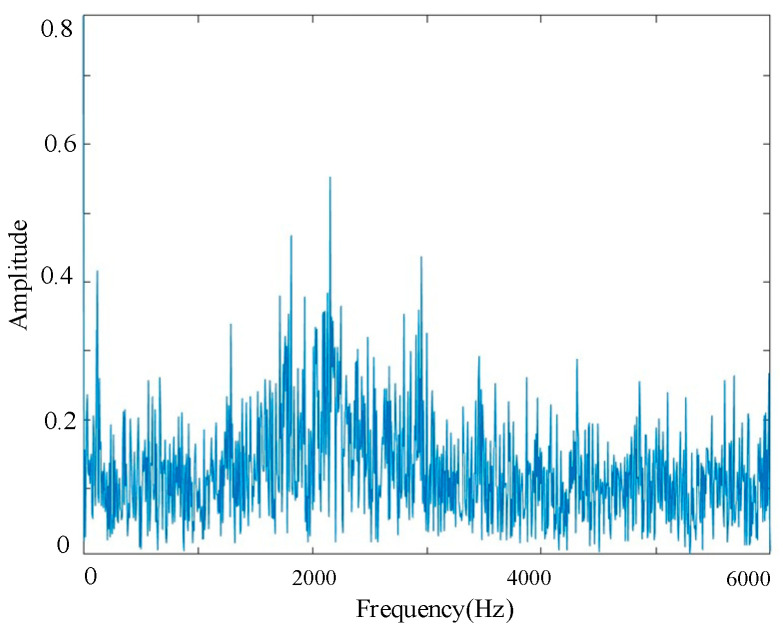
Frequency spectrum of broken tooth signal.

To investigate the frequency–domain characteristics of gear faults, a representative set of signals with broken-tooth faults is selected as a case study. Fast Fourier Transform (FFT) is applied to reveal the frequency characteristics of the signal. As shown in Figure 4, the main frequency components are concentrated within the range of 0–3000 Hz, with a prominent spectral peak observed around 2200 Hz. This peak is typically associated with the periodic impacts caused by broken teeth, which introduce abrupt changes in meshing force and excite strong vibration responses. Notably, under the current gear parameters and rotational speed, 2200 Hz corresponds to a harmonic of the gear mesh frequency (GMF), making it a critical indicator for identifying broken-tooth faults.

**Figure 5:**
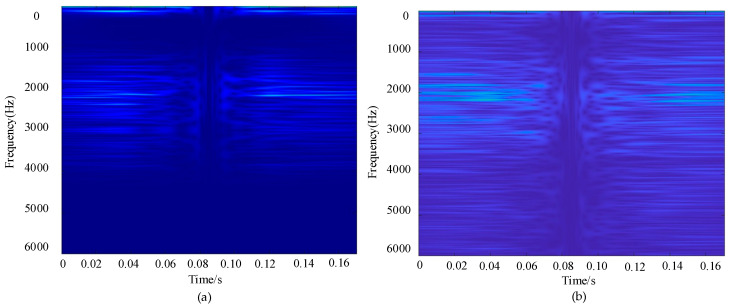
Comparison of time-frequency spectrograms. (**a**) Improved S-transform; (**b**) standard S-transform.

To further evaluate the performance of the proposed diagnostic method under complex operating conditions, background noise of a certain intensity was artificially added to the original vibration signals during the experimental phase. On this basis, both the standard S-transform and the improved S-transform were applied for time–frequency analysis and comparison. As shown in Figure 5a, the improved S-transform provides a clearer representation of the signal’s frequency characteristics within the 0–3000 Hz range, particularly enhancing the visibility of the fault-related frequency component around 2200 Hz while effectively suppressing background noise. In contrast, the time–frequency map obtained using the standard S-transform, as shown in Figure 5b, exhibits an extended frequency range up to 5000 Hz, in which the fault characteristic frequency is partially obscured and significantly affected by background noise. Therefore, the improved S-transform not only preserves critical fault features, but also reduces the interference of background noise, thereby enhancing the time–frequency representation of the signal and providing stronger support for subsequent fault diagnosis.

### 4.2. Fault Diagnosis Analysis

#### 4.2.1. Dataset Construction

To fully leverage the advantages of the proposed integration of the improved S-transform and DSCNN, a dataset was constructed based on the time-frequency representations obtained from 1000 collected signal samples using the improved S-transform. The dataset includes five conditions, comprising one normal state and four different types of gear faults, with 200 samples per class to ensure class balance. To enhance the model’s generalization ability, the dataset was divided into a training set and a testing set in an 8:2 ratio, with 800 samples used for training and 200 samples for testing. The processed training data were subsequently fed into the DSCNN for fault classification. The construction and partitioning details of the fault diagnosis dataset are presented in Table 1.

#### 4.2.2. Model Parameter Settings

The experiments were conducted on a system equipped with an Intel Core i5-12400F CPU and an AMD RX series GPU. During network training, the Adam optimizer was employed with the following hyperparameters: batch size of 32, maximum of 50 training epochs, gradient clipping threshold set to 1, initial learning rate of 0.001, and a stepwise learning-rate decay strategy that reduces the learning rate by a factor of 0.1 every 10 epochs. An L2 regularization coefficient of 1 × 10^−4^ was applied. Training data were randomly shuffled every epoch, and validation was performed every 30 iterations. The training process dynamically selected CPU or GPU computation and displayed real-time training progress. By appropriately setting hyperparameters such as batch size and learning rate, the training process can be stabilized.

#### 4.2.3. Diagnostic Results Analysis

**Figure 6:**
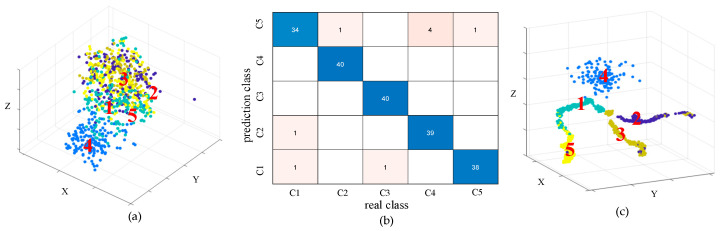
Confusion matrix and t-SNE visualization of the DSCNN Model. (**a**) t-SNE of the input layer; (**b**) confusion matrix; (**c**) t-SNE of output layer.

After training, the trained model was evaluated on the test set, and both the confusion matrix and t-SNE visualizations at different layers were plotted. As shown in the t-SNE distribution of the input data layer in Figure 6a, distinct fault categories did not form clear clusters in the original feature space; instead, the distributions of different classes were mixed and boundaries were blurred, indicating the inherent complexity and poor separability of the gear fault data. After model training, the t-SNE visualization of the output layer in Figure 6c demonstrates a clear separation between classes, suggesting that the model progressively extracted highly discriminative deep features during the learning process. This transformation also confirms the effectiveness of the improved S-transform combined with the depthwise separable network architecture in feature extraction and pattern recognition. Furthermore, the confusion matrix in Figure 6b shows that the model achieves high recognition accuracy across five fault categories (tooth crack, tooth breakage, tooth wear, tooth deficiency, and normal), particularly with near-zero misclassification between tooth crack and normal gear states. The model attained a classification accuracy exceeding 98% on the test set, validating the superior performance of the proposed method in gear fault diagnosis and providing reliable technical support for fault detection under complex operating conditions.

To gain deeper insights into the model’s decision-making process, this study employs t-Distributed Stochastic Neighbor Embedding(t-SNE) and Gradient-weighted Class Activation Mapping (Grad-CAM) to visualize the features extracted from different convolutional layers during training. Specifically, t-SNE and Grad-CAM analyses were conducted on four convolutional layers (Conv2, Conv3, Conv4, and Conv5) of the deep separable convolutional network to investigate the characteristics learned by the model at various depths.

**Figure 7:**
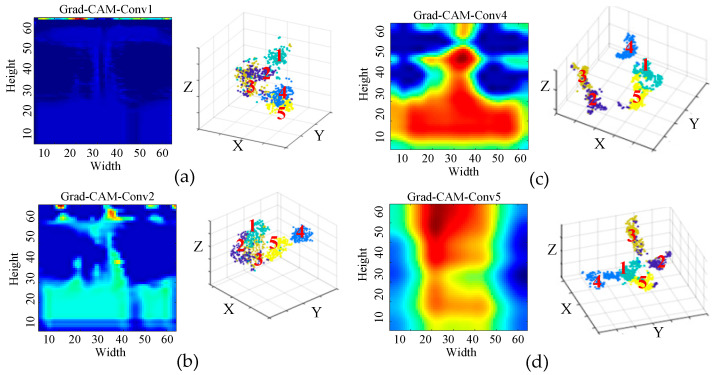
Visualization results of the network feature extraction layers. (**a**) Grad-CAM-Conv1; (**b**) Grad-CAM-Conv2; (**c**) Grad-CAM-Conv4; (**d**) Grad-CAM-Conv5.

Figure 7 presents the visualization results of the network feature extraction layers. The t-SNE visualization of the Conv2 layer reveals that the data distribution at this stage is relatively disordered, with the original fault features difficult to distinguish directly at the input phase, as there is significant overlap among different classes. Analysis of the Grad-CAM results for the Conv3 layer indicates that the model has begun to focus on more prominent features within the 0–3000 Hz frequency range, although considerable irrelevant information remains. Meanwhile, the t-SNE results of both Conv2 and Conv3 layers show an improved, but still incomplete, separation of fault categories, suggesting that the features within the 0–3000 Hz range primarily correspond to shallow convolutional features. These features can serve as auxiliary cues for manual fault discrimination, but do not represent the definitive basis for classification.

Further analysis of the Conv4 and Conv5 layers reveals a significant improvement in clustering performance as visualized by t-SNE, with clearer boundaries between different classes. This indicates that the model has learned more discriminative features through deeper convolutional layers. Meanwhile, the Grad-CAM results demonstrate that the activation regions have become more stable and closely related to the final fault classification, suggesting that the high-level features extracted by the deeper convolutional layers constitute the core basis for the model’s fault diagnosis decisions.

In summary, although the attention in the early layers (e.g., approximately 0–3000 Hz) is partly interpretable and aligns with known signal characteristics, the deeper attention patterns are less directly explainable, reflecting the complexity and abstraction of the learned features. This transition from interpretable low-level features to abstract high-level representations is consistent with the hierarchical nature of deep learning models. While deep features are more effective for classification, they pose challenges for physical interpretability, representing a common trade-off in deep neural networks.

#### 4.2.4. Imbalanced Class Experiment and Analysis

In this imbalanced training experiment, the dataset consists of five classes, each containing 200 samples. To simulate the common small-sample problem encountered in real-world scenarios, the training set includes 10 samples of tooth wear, 15 samples of tooth breakage, 12 samples of tooth deficiency, 80 samples of tooth crack, and 160 samples of normal, with the remaining samples used for testing. This setup reduces the number of training samples in the first three classes to 5–7.5% of their total, aiming to replicate the realistic scenario where majority classes are sufficiently represented while minority classes are scarce, thereby evaluating the model’s robustness under small-sample conditions. Table 2 presents the classification performance metrics for each gear fault category.

The experimental results indicate a certain degree of overall performance degradation of the model. Although tooth wear, tooth breakage, and tooth deficiency are typical small-sample categories, their F1-scores still reach 0.91, 0.89, and 0.85, respectively, demonstrating that the model retains basic discriminative ability for these categories. This outcome can be partly attributed to the improved S-transform’s capability in frequency–domain feature extraction, enabling the model to extract relatively effective discriminative information from limited samples. However, data imbalance significantly affects the model’s decision boundaries. Despite the normal class having the largest number of training samples (160), its precision is only 0.5063, although recall is 1.0, indicating that the model tends to classify samples as normal, leading to a high number of false positives. This phenomenon reflects a “majority class dominance” bias, a common issue in imbalanced classification. In contrast, the tooth crack class, with a moderate training sample size (80), exhibits the most stable performance, achieving precision, recall, and F1-score of 0.9912, 0.9932, and 0.9897, respectively. This suggests that when the sample size is moderate, the model’s recognition ability is relatively robust, with minimal fluctuation in classification performance. Overall, under this extreme imbalance setting, the model attains a macro-average F1-score of 0.8639 and an overall accuracy of 88.52%, showing a noticeable decline compared to training with balanced data. These results highlight that although the model possesses some small-sample adaptability, severe class imbalance can bias the decision boundaries toward majority classes, adversely affecting overall recognition performance. Future work may consider incorporating few-shot learning, data augmentation, or transfer learning strategies to further mitigate performance degradation caused by sample imbalance.

### 4.3. Comparative Experiment

#### 4.3.1. Analysis of Recognition Results Using Different Transformation Methods

**Figure 8:**
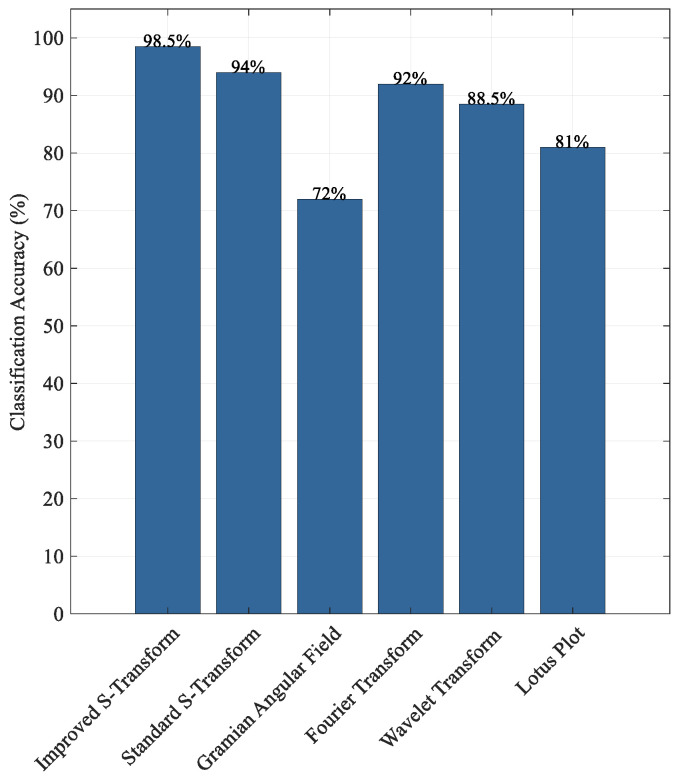
Comparison of fault identification results using different time–frequency methods.

To validate the effectiveness of time–frequency representation images as model inputs for fault diagnosis, this study applied WT, Fourier Transform (FT), and Gramian Angular Field (GAF) methods to the noisy signals. The generated time–frequency images were then used as inputs for diagnostic analysis. As shown in Figure 8, the proposed IST method achieved the best performance in fault classification, with an accuracy of 98.5%, significantly outperforming other common time–frequency transformation methods. The standard ST achieved an accuracy of 94%, while FT and WT reached 92% and 88.5%, respectively. In contrast, the Lotus plot (81%) and GAF (72%) exhibited relatively lower performance, indicating weaker feature extraction capability under noisy conditions. Furthermore, the fault identification results demonstrate that frequency–domain or time–frequency domain transformation methods generally outperform pure time–domain images, further confirming the advantages of the proposed method in terms of robustness and classification accuracy.

#### 4.3.2. Visualization-Based Comparison of Time–Frequency Transformation Methods

**Figure 9:**
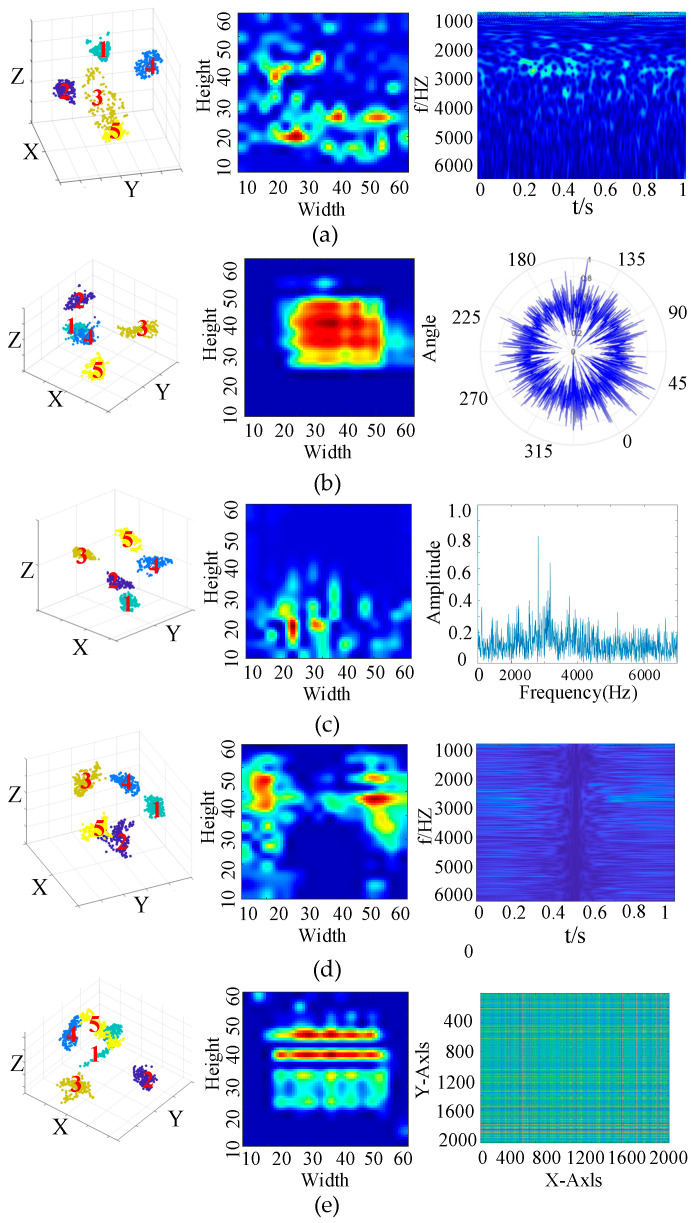
Analysis of features and decision mechanisms of different time–frequency methods in fault identification. (**a**) visualization of Wavelet Transform; (**b**) visualization of Lotus Plot; (**c**) visualization of Fourier Transform; (**d**) Visualization of standard S-transform; (**e**) visualization of Gramian Angular Field.

As shown in Figure 9, a comprehensive analysis of Grad-CAM and t-SNE visualizations for different signal transformation methods reveals significant differences in feature attention and feature distribution. Firstly, the t-SNE visualization indicates clear differences in feature separability among the methods. Although most transformation methods exhibit relatively clear overall feature distributions, some methods (such as the Gramian Angular Field) still show class overlap, suggesting insufficient discriminative capability. In contrast, the proposed S-transform method demonstrates stronger inter-class separability in the low-dimensional projection space, further validating its superiority in feature extraction.

From the Grad-CAM visualizations, various time–frequency transformation methods exhibit distinct patterns of model attention. The Wavelet Transform, with its multi-scale analysis capability, enables the model to process features across multiple frequency bands. Although differences in the high-frequency region are less pronounced, the attention distribution remains interpretable and aligns with known structures of mechanical vibration signals, demonstrating how Grad-CAM provides insights into fault localization at different frequency scales. In comparison, the Lotus plot produces a tightly focused attention region. While this may indicate effective localization, the attention is restricted to a narrow area with limited physical justification. The Fourier Transform highlights attention to high-frequency bands, especially around 2200 Hz, which are typically associated with fault harmonics. Grad-CAM clearly emphasizes this correspondence, illustrating how the model leverages frequency–domain information. The standard S-transform generates more stable and concentrated attention in time–frequency regions related to faults, thereby achieving better focus and interpretability. The Gramian Angular Field produces highly dispersed attention maps; in this case, Grad-CAM reveals that the model’s focus is scattered and lacks clear alignment with known diagnostic features. This makes it difficult to understand how the model arrives at its predictions, despite acceptable model performance.

In summary, not all features attended to by the model are clearly interpretable. Different time–frequency transformation methods exhibit distinct visualization outcomes: some generate attention maps consistent with domain knowledge, enhancing trust in the model’s decision rationale, while others produce abstract or dispersed attention regions, weakening the clarity of the diagnostic logic. In this context, Grad-CAM serves not only as a visualization tool, but also as an interpretability analysis technique that helps assess whether the model genuinely focuses on diagnostically relevant signal regions, providing an important basis for evaluating model reliability.

#### 4.3.3. Comparison of Diagnostic Performance Among Models

To evaluate the fault diagnosis capability of the proposed model under the complex operating conditions of the shearer rocker arm gear, this study compares the performance of multiple deep learning models on the fault recognition task under the same conditions. The models compared include DeepCNN (DCNN), InceptionV3, ResNet, PyramidCNN (PCNN), and the proposed model. Among these, DCNN employs a simple stacked structure of multiple convolutional and pooling layers. InceptionV3 introduces multi-scale parallel convolutional branches to enhance feature representation. ResNet mitigates the vanishing gradient problem through residual connections. PCNN adopts a pyramid structure that deepens layer by layer to strengthen abstraction capabilities.

**Figure 10:**
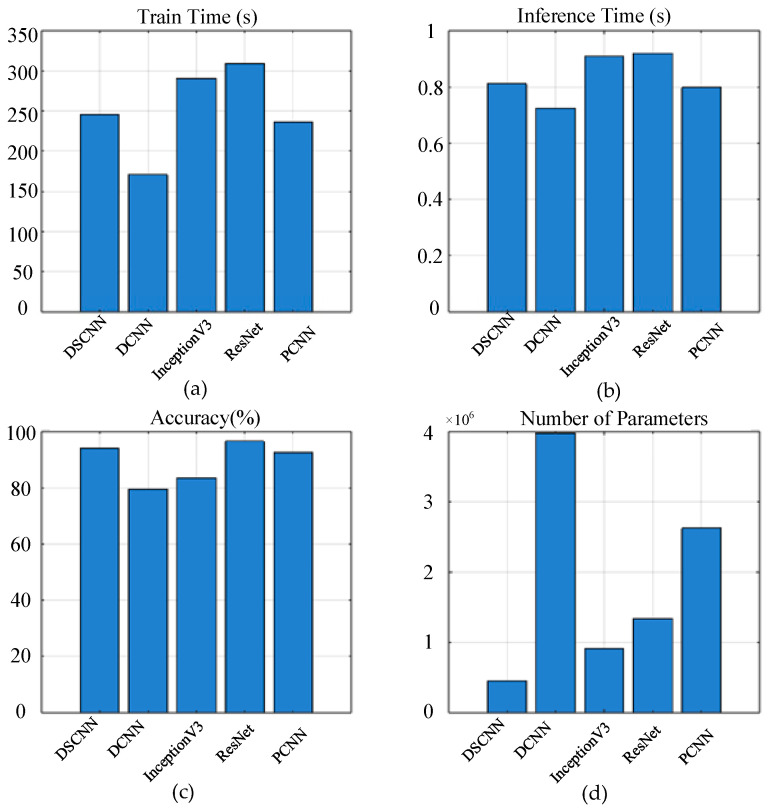
Analysis of diagnostic results of different models. (**a**) Train time; (**b**) inference time; (**c**) accuracy; (**d**) number of parameters.

To ensure a fair comparison, the input size of all models was standardized to 64 × 64 × 3, and the output was set to five classes. All models were evaluated on the same training dataset and using consistent evaluation metrics, with unified training strategies including the number of epochs, optimizer, learning rate, and batch size. Although different networks employ their respective classic design approaches (such as the stacked structure of DCNN, residual connections of ResNet, and multi-scale parallel branches of InceptionV3), efforts were made to maintain similar network depths and numbers of convolutional layers across models. This ensured that their feature extraction capabilities were roughly on the same level, minimizing performance bias caused by architectural differences. For example, all models contain approximately five to seven convolutional layers; some models, such as InceptionV3 and the proposed model, utilize parallel or channel-expansion structures, but the overall parameter count was controlled within a reasonable range. The diversity of feature extraction strategies among these models provides a representative basis for subsequent performance evaluation. Figure 10 presents the performance results of each model in the fault diagnosis task for the shearer rocker arm gear, including key metrics such as training time, inference time, accuracy, and parameter count.

In terms of accuracy, the ResNet model achieved the highest accuracy of 96.50%, demonstrating the advantage of its residual structure in extracting complex features. The PCNN also attained a relatively high accuracy, of 92.50%, indicating that its multi-level pyramid architecture effectively enhances feature representation. The proposed model maintained a high accuracy of 94.00% while achieving a better balance between performance and efficiency with the lowest parameter count (457,509) and a relatively short inference time (0.8112 s). Regarding training time, DCNN required the shortest duration (170.65 s), but its recognition performance was relatively weak, failing to adequately capture the complex features of the shearer rocker arm gear faults. InceptionV3 incurred higher training and inference costs with only limited accuracy improvement, underperforming compared to ResNet and PCNN. Overall, although ResNet and PCNN slightly outperformed in accuracy, they demand larger model sizes and longer inference times. In contrast, the proposed model demonstrates superior overall performance by achieving fewer parameters, faster inference speed, and high accuracy. Therefore, the proposed method exhibits better applicability for fault diagnosis of shearer rocker arm gears.

## 5. Conclusions

(1) The improved S-transform effectively enhances the fault-related frequency components of the gear vibration signals. Compared with the original S-transform, it produces clearer frequency–domain features and reduces the interference of background noise on fault characteristics. Furthermore, the introduced depthwise separable structure improves computational efficiency, reducing model training time, parameter size, and inference overhead, while maintaining high accuracy. This makes the method more suitable for fault diagnosis of shearer arm gearboxes under complex working conditions.

(2) Frequency–domain information contributes significantly to improving fault recognition performance. When one-dimensional time-series signals are transformed into two-dimensional frequency or time–frequency images and fed into 2D models, the recognition accuracy is generally higher than that based on raw time–domain signals. Notably, even under certain background noise levels, the frequency-based approach can still maintain classification accuracy above 90%.

(3) The interpretability analysis based on Grad-CAM reveals that the model focuses on features that are closely aligned with the physical mechanisms of gear faults, thereby enhancing the traceability and trustworthiness of the diagnostic process. However, some deep-layer features remain abstract and cannot be directly linked to specific physical phenomena, suggesting that future studies should integrate domain knowledge to further improve the explainability and transparency of the model, helping to demystify its “black-box” nature.

(4) Although the proposed method achieves promising classification performance on a controlled and balanced dataset, its generalization ability under imbalanced data distributions and variable operating conditions has not been fully evaluated. Future work will explore the integration of transfer learning and few-shot learning techniques, to enhance the model’s adaptability and robustness across different equipment and complex environments. Additionally, domain-informed interpretability strategies will be investigated to further improve model credibility and facilitate real-world deployment.

## Figures and Tables

**Table 1:** Construction and partitioning of the fault diagnosis dataset.

Fault ID	Fault Type	Speed (HZ)	Training Set	Test Set	Total Samples	Label
C1	tooth wear	30	160	40	200	1
C2	tooth crack	30	160	40	200	2
C3	tooth breakage	30	160	40	200	3
C4	tooth deficiency	30	160	40	200	4
C5	normal	30	160	40	200	5
——	sum	——	800	200	1000	——

**Table 2:** Classification performance metrics of gear fault categories.

Fault ID	Fault Type	Precision	Recall	F1-Score
C1	tooth wear	0.9645	0.8579	0.9081
C2	tooth crack	0.9912	0.9932	0.9897
C3	tooth breakage	0.8600	0.9297	0.8935
C4	tooth deficiency	0.9355	0.7713	0.8455
C5	normal	0.5063	1.0000	0.6723

## Data Availability

The data are available on reasonable request from the author.
